# Construction of circadian clock signature for tumor microenvironment in predicting survival of esophageal squamous cell carcinoma

**DOI:** 10.3389/fimmu.2026.1738892

**Published:** 2026-02-12

**Authors:** Yiping Xiang, Xuelian Cui, Zhe Cui

**Affiliations:** 1 Department of Pathology, Tianjin Medical University Cancer Institute and Hospital, National Clinical Research Center for Cancer, Tianjin Key Laboratory of Digestive Cancer, Tianjin’s Clinical Research Center for Cancer, Tianjin, China; 2 Division of Hematology and Transfusion Medicine, Tianjin Medical University Baodi Hospital, Tianjin, China

**Keywords:** circadian clock, esophageal squamous cell carcinoma, immune infiltration, prognostic model, TCGA

## Abstract

**Background:**

Esophageal squamous cell carcinoma (ESCC) is a distinct subtype of esophageal cancer (EC). Research indicates that circadian clock genes (CCGs) in human ESCC are dysregulated. However, the significance of CCGs in ESCC prognosis remains ambiguous. This study sought to establish a complete signature of ESCC-specific differentially expressed CCGs (DE-CCGs) associated with prognosis, tumor growth, and immunological infiltration.

**Methods:**

Differentially expressed genes (DEGs) between normal and ESCC samples in TCGA database and the GSE23400 dataset were intersected with CCGs to obtain DE-CCGs. The prognosis-related DE-CCGs were discerned to develop a risk model using univariate Cox regression and LASSO regression analyses in TCGA-ESCC. The accuracy of the model was validated using risk and overall survival profiles.

**Results:**

Seven DE-CCGs (CST3, C1QBP, TTF2, EGFR, CDKN2A, PFAS, TRRAP) were identified in TCGA-ESCC, which were correlated with unfavorable ESCC prognosis. The immune infiltration analysis revealed that High-risk ESCC patients displayed enhanced tumor infiltration. And the combination of CST3 and PD-L1 expression may serve as a potential marker for predicting prognosis of ESCC patients. Moreover, in Vitro experimental models, CST3 expression was markedly elevated in tumor cells and associated with ESCC growth.

**Conclusions:**

This research illustrated the prognostic significance of seven DE-CCGs for ESCC patients based on tumor progression and immune infiltration. And the CST3 may serve as an independent prognostic biomarker and a potential therapeutic target for ESCC.

## Graphical Abstract

**Figure:**
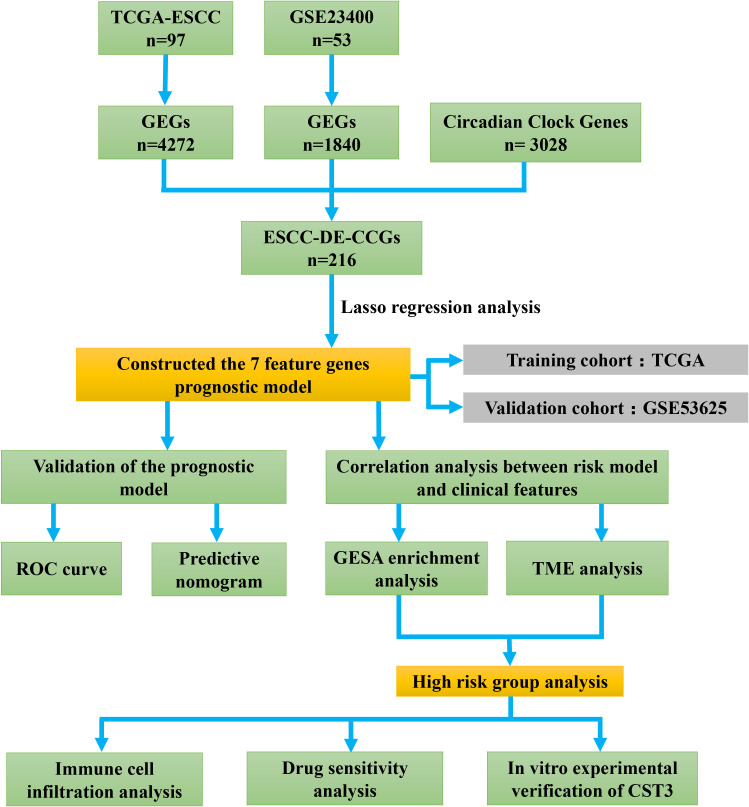

## Introduction

Esophageal cancer (EC) ranks as the eighth most prevalent malignancy and the sixth chief cause of cancer-related death worldwide (1). Esophageal squamous cell carcinoma (ESCC) is the predominant subtype, accounting for around 84% of all EC cases (2). Although targeted therapy and immunotherapy have addressed the limitations of EC treatment and are increasingly becoming first-line therapeutic options, clinical outcomes for EC remain unsatisfactory (3, 4). Given that relevant immunotherapies for EC remain in the nascent phase of investigation (5), there is a pressing need for innovative antitumor agents and therapeutic strategies for advanced ESCC, alongside the urgent necessity to establish new diagnostic techniques for tracking cancer development.

Circadian clock genes (CCGs) exhibit rhythmic expression following the day-night cycle, regulating several physiological processes, such as blood pressure, hormone production, sleep, and immunological functions (6, 7). Accumulating research has stated that the circadian clock is essential for cancer development and progression through complicated mechanisms, which showed that CCGs may be new targets for cancer diagnosis and treatment. The tumor microenvironment (TME) has emerged as a critical regulator of tumor progression and therapeutic resistance, driving the development of numerous TME-targeted therapies (8). Emerging evidence suggests clock genes coordinately modulate multiple TME remodeling processes, including angiogenesis, tumor-promoting inflammation, and immune evasion (9). For example, the expression of clock genes (e.g., CLOCK, BMAL1, and Cry1) in kidney renal clear cell carcinoma (KIRC) and breast cancer demonstrate strong correlations with the infiltration of macrophages, neutrophils, and dendritic cells (10, 11). Another independent group also revealed that the expression of BMAL1 is identified as a regulator of T-cell functional states, controlling T-cell activation/differentiation markers and exhaustion-associated checkpoints (CTLA4, PD-1, and PD-L1) in metastatic melanomas (12). Given the substantial influence of the TME in determining the efficacy of cancer immunotherapy, circadian rhythms may modulate treatment sensitivity by regulating immune cell function and inflammatory responses. Currently, CCGs concerning the TME have not been comprehensively investigated in ESCC, notwithstanding advances in biomarker research in the TME. Thus, integrating the mechanisms of CCGs with TME research may provide novel insights for prognostic stratification in ESCC.

Consequently, this work sought to identify differentially expressed CCGs (DE-CCGs) of ESCC through bioinformatics techniques and to develop a risk model for assessing the prognostic significance and relevance to the TME of characteristic genes for ESCC. The relationships of the specific prognostic markers CST3 with immune infiltration and tumor progression were further examined to pinpoint ESCC-specific prognostic biomarkers and treatment targets.

## Materials and methods

### Data sources

The expression data and clinical traits of TCGA-ESCC including 13 normal and 97 ESCC samples were obtained from TCGA database (https://portal.gdc.cancer.gov/). GSE23400 and GSE53625 dataset was acquired from the GEO database (https://www.ncbi.nlm.nih.gov/geo/). Moreover, 3028 CCGs were downloaded from the Gene Cards database (https://www.genecards.org/).

### Identification of DE-CCGs between ESCC and normal samples

DESeq2 packages was utilized for DEG analysis in TCGA-ESCC with p < 0.05 as the screening threshold, while limma R packages were utilized in GSE23400 cohorts. Volcano plots generated with the ggplot2 R package visualized DEG expression meeting the statistically significant threshold “adjusted P <0.05 and | log2FC | >1”.

WGCNA was leveraged to discern highly correlated gene modules with the WGCNA R package (13). The soft thresholding parameter was 5 for TCGA-ESCC and 6 for GSE23400 to develop a gene network and compute coexpression similarity and adjacency, which were then converted into a topological overlap matrix (TOM). The modules were then grouped through hierarchical clustering. The modules that were closely linked with clinical features were recognized. Ultimately, highly correlated DEGs were intersected with CCGs to derive DE-CCGs, which were illustrated in a Venn diagram.

### Construction of a prognostic risk model

Univariate Cox analysis was leveraged to pinpoint DE-CCGs related to ESCC prognosis, which were visualized via the survival and forest plot R packages. Genes with p < 0.05 were included in LASSO analysis to prevent overfitting utilizing the glmnet R package (14). The expression levels of each gene were multiplied with its corresponding weighting coefficients to calculate the risk score: Risk score = (expression of CST3* 0.54179124) + (expression of C1QBP* -0.298329668) + (expression of TTF2* -0.121834423) + (expression of EGFR* -0.068621876) + (expression of CDKN2A* -0.262690427) + (expression of PFAS* -0.143824307) + (expression of TRRAP* -0.357097173). 97 TCGA-ESCC samples were categorized into high-risk and low-risk groups based on the median risk score. The predictive power of the model was confirmed by Kaplan–Meier analysis utilizing the survminer R package.

### Validation of the prognostic risk model

The expression of prognostic genes was shown in a heatmap with the ComplexHeatmap R package. A nomogram was developed to forecast the overall survival (OS) based on risk score and clinicopathologic traits (e.g., age, sex, and stage) with the rms R package. Endpoints of 1, 2, and 3 years were established, and the prognostic performance was appraised through receiver operating characteristic (ROC) curves utilizing the pROC R package.

### Enrichment analysis

Biological functional enrichment was examined through gene set enrichment analysis (GSEA). The threshold was established at p < 0.05. Results were visualized with the clusterProfiler R package.

### Immune infiltration analysis

R package-IOBR was selected to determine the links between prognostic genes, risk scores, and tumor-infiltrating immune cells (TIICs). Eight methods (CIBERSORT, ESTIMATE, TIMER, IPS, quanTIseq, MCPCounter, xCell, and EPIC) were utilized for scoring immune infiltration. Additionally, immune cell types over-represented in the TME were identified by single sample GSEA (ssGSEA) (15). The Tumor Immune Estimation Resource (TIMER, https://cistrome.shinyapps.io/timer/) was utilized to quantify immune cell infiltration from TCGA (16).

### Drug sensitivity analysis of CST3

Data on gene susceptibility to drugs were sourced from the CellMiner database (17). Pearson’s correlation analysis was applied between CST3 expression and drug sensitivity. Results were visualized with the ggpubr and ggplot2 R package.

### Patients and tissues

Tumor tissues and adjacent normal tissues were collected from 102 ESCC patients undergoing primary surgical treatment in Tianjin Medical University Cancer Institute and Hospital. None of the patients had received prior treatment or had a history of other malignancies. All patients underwent accurate surgery base on their clinical examinations combined with pathological diagnosis. This work was conducted in compliance with the Declaration of Helsinki and approved by the ethics committee of Tianjin Medical University Cancer Institute and Hospital (Ethics Approval Number: bc20252428). Written informed consent was obtained from all participants.

### Evaluation of IHC

IHC results were evaluated by two pathologists in a blinded manner. Ambiguous cases were examined using a multiheaded microscope and discussed by two pathologists until a consensus was achieved. CST3 expression was mainly located in the cytoplasm of ESCC cells, and PD-L1 staining was located on the cell membrane or cell cytoplasm. CST3 and PD-L1 staining results were ascertained by randomly selecting 5 high-power fields (40×10), with 100 tumor cells examined in each high-power field. The percentage of positive cells was computed, with <10%, 10%~24%, 25%~50%, >50% scored as 0, 1, 2, and 3, respectively. The staining intensity ranged from no positive staining, light yellow, yellow, and brown, and scored from 0 to 3. The two scores were multiplied, with <3 scores classified as negative and ≥3 classified as positive. Necrotic regions were omitted from the evaluation.

### Antibodies and reagents

Antibodies applied in Western blot included CST3 (1:1000, ab109508, Abcam) and β-actin (1:2000 dilution, AC026, abclonal). Antibodies used in IHC included CST3 (1:50, ab109508, Abcam) and PD-L1(1:50 dilution, ab205921, Abcam). CD274 (PD-L1, B7-H1) monoclonal antibody (MIH1) was purchased from eBioscience. The sequence for CST3 siRNA was 5’ - GGACUUGAUGAUGAAGAAATT - 3’.

### Western blotting

Western blot was conducted to test protein expression in the indicated ESCC cells as described previously by us (18).

### Cell proliferation assay

Cell proliferation was testified via CCK-8 and plate colony formation assays as described previously by us (18). The assays were performed in triplicate.

### Statistical analysis

Statistical analyses were done in R software 4.3.2. Data are delineated as means ± standard deviation (SD). Qualitative variables were compared using the student’s t-test or χ2-test. P < 0.05 implied significance.

## Results

### Identification of the highly correlated gene modules between ESCC and normal samples

**Figure 1:**
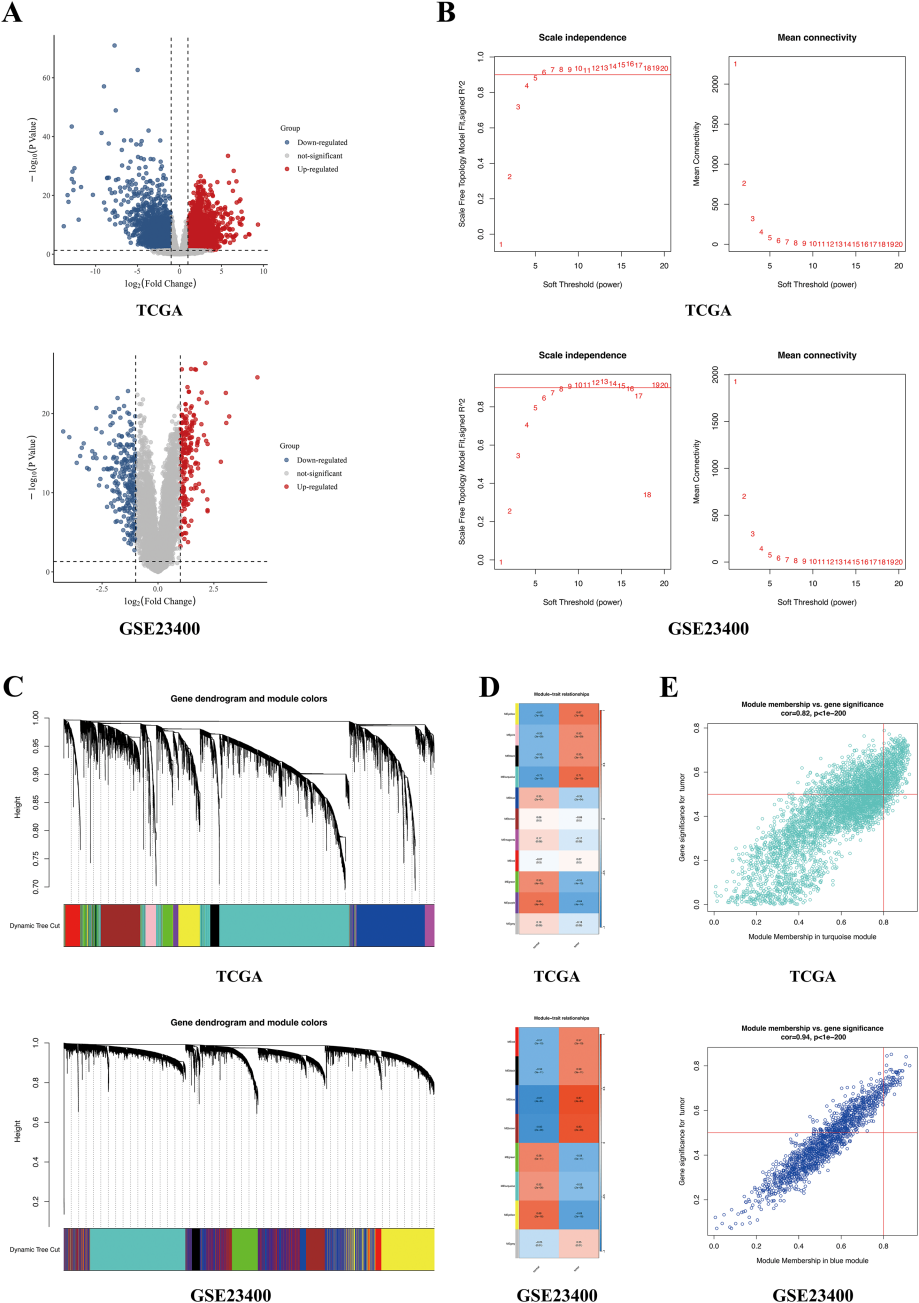
WGCNA algorithm screening of DEGs in the TCGA and GSE23400 dataset. **(A)** The volcano map of DEGs. The blue and red dots represent significantly lower and higher DEGs, respectively; the black horizontal line represents p<0.05, and the two vertical lines represent | log2FC | >1. **(B)** Analysis of the scale-free fit index for various soft-thresholding powers and the mean connectivity for various soft-thresholding powers. **(C)** The dendrogram of all genes is clustered based on a dissimilarity measure (TOM). **(D)** The heatmap shows eleven and eight modules were identified between normal and tumor in TCGA and GSE23400 respectively. Red and blue represent a positive/negative correlation between MEs and samples. **(E)** Gene correlation scatter plot of the turquoise module and blue module. The x-axis represents module membership (MM). The y-axis represents gene significance (GS). The p-value is <0.0001. (MM, i.e., the correlation between the genes and modules) (GS, i.e., the correlation between the genes and clinical traits).

Compared with normal samples, 2491 DEGs were upregulated and 2686 downregulated in TCGA, whereas 243 were upregulated and 203 downregulated in GSE23400 (**Figure 1A**). To investigate the strongly correlated genes among these DEGs, WCGNA was used to identify the highly correlated gene modules with the most suitable soft-thresholding power (**Figure 1B**). Then, all gene modules were categorized based on the TOM by hierarchical clustering (**Figure 1C**). Eleven modules were discovered in the TCGA-ESCC cohort and eight modules were discovered in the GSE23400 cohort (**Figure 1D**). Among these gene modules, the turquoise module in TCGA-ESCC (the coefficient = 0.71 and p = 3e-18) and the blue module in GSE23400 cohorts (the coefficient = 0.87 and p = 4e-34) were connected with tumor occurrence. Furthermore, the gene significance and module membership were highly correlated in the turquoise and blue modules, suggesting a strong association between cancers and genes in these modules (**Figure 1E**). Finally, we extracted 4272 genes from the turquoise module and 1840 genes from the blue modules for further analysis. The intersection of these genes revealed 339 candidate core genes.

### Construction of CCGs-related prognostic risk model in ESCC

**Figure 2:**
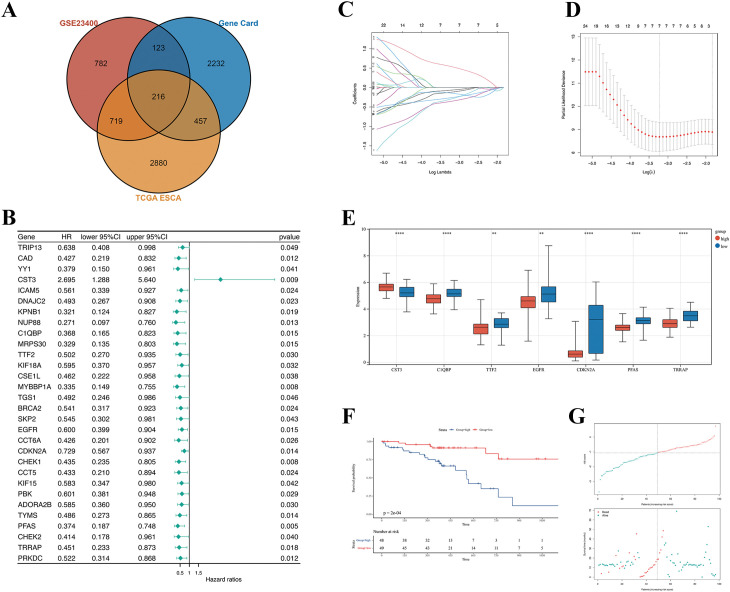
Construction of a CCGs-related prognostic risk model. **(A)** Venn diagram of overlapping genes in TCGA, GSE23400, and CCGs. **(B)** Forest plot of the 30 candidate prognostic genes. The 95% confidence interval of the Hazard ratio is displayed. Genes with a hazard ratio > 1 are detrimental prognostic genes. The p-value is <0.05. **(C)** Coefficient curve. Different colors represent different genes. **(D)** The minimum lambda of the lasso model was selected via 10 folds of cross-validation. Lambda was determined when the partial likelihood of deviance was smallest. **(E)** Boxplot of the expression of prognostic genes in the different risk groups. The patients were divided according to their median risk score. **(F)** OS of the different risk groups. **(G)** Risk curve of risk score distributions and survival times for the different risk groups. Upper panel: The x-axis represents the number of patients and the y-axis represents the risk score. Lower panel: The x-axis represents the number of patients and the y-axis represents the survival time of the patients. **P < 0.01; **** p < 0.0001.

To pinpoint DE-CCGs, we crossover 3028 CCGs from the Gene Cards database with the highly correlated DEGs in TCGA-ESCC and GSE23400 and finally screened 216 target genes (**Figure 2A**). Subsequently, Univariate Cox analysis recognized 30 genes with p < 0.05 (**Figure 2B**). Following that, LASSO algorithm, based on the minimum standard, recognized seven candidate core genes (CST3, C1QBP, TTF2, EGFR, CDKN2A, PFAS, TRRAP) to establish a prognostic signature (**Figures 2C–D**). The risk score of each ESCC patient was gauged, and patients were categorized into high-risk (HR) and low-risk (LR) groups according to the median risk scores. Prognostic genes were markedly downregulated in HR patients compared to the LR group, except CST3, which was considerably increased (**Figure 2E**). In addition, CST3 was negatively linked with the other six genes, denoting that the seven genes had a significant functional similarity in ESCC (**Supplementary Figure 1**). Kaplan–Meier analysis elicited that HR patients had reduced survival probability in comparison to the LR group (**Figure 2F**; P < 0.001). All prognostic genes were considerably linked with poor survival probability (**Supplementary Figure 2**). The distribution of risk scores and survival status are plotted (**Figure 2G**). Furthermore, the overall survival, survival time and gene expression profiles of the prognostic genes in the test dataset were consistent with those in the training dataset (**Supplementary Figure 3**). Collectively, we constructed a CCGs-related prognostic risk model including seven ESCC-specific prognostic genes, revealing that CST3 is the only oncogene significantly overexpressed in ESCC, whilst the other genes are under-expressed as tumor suppressor genes.

### Performance of CCG-related prognostic signature

**Figure 3:**
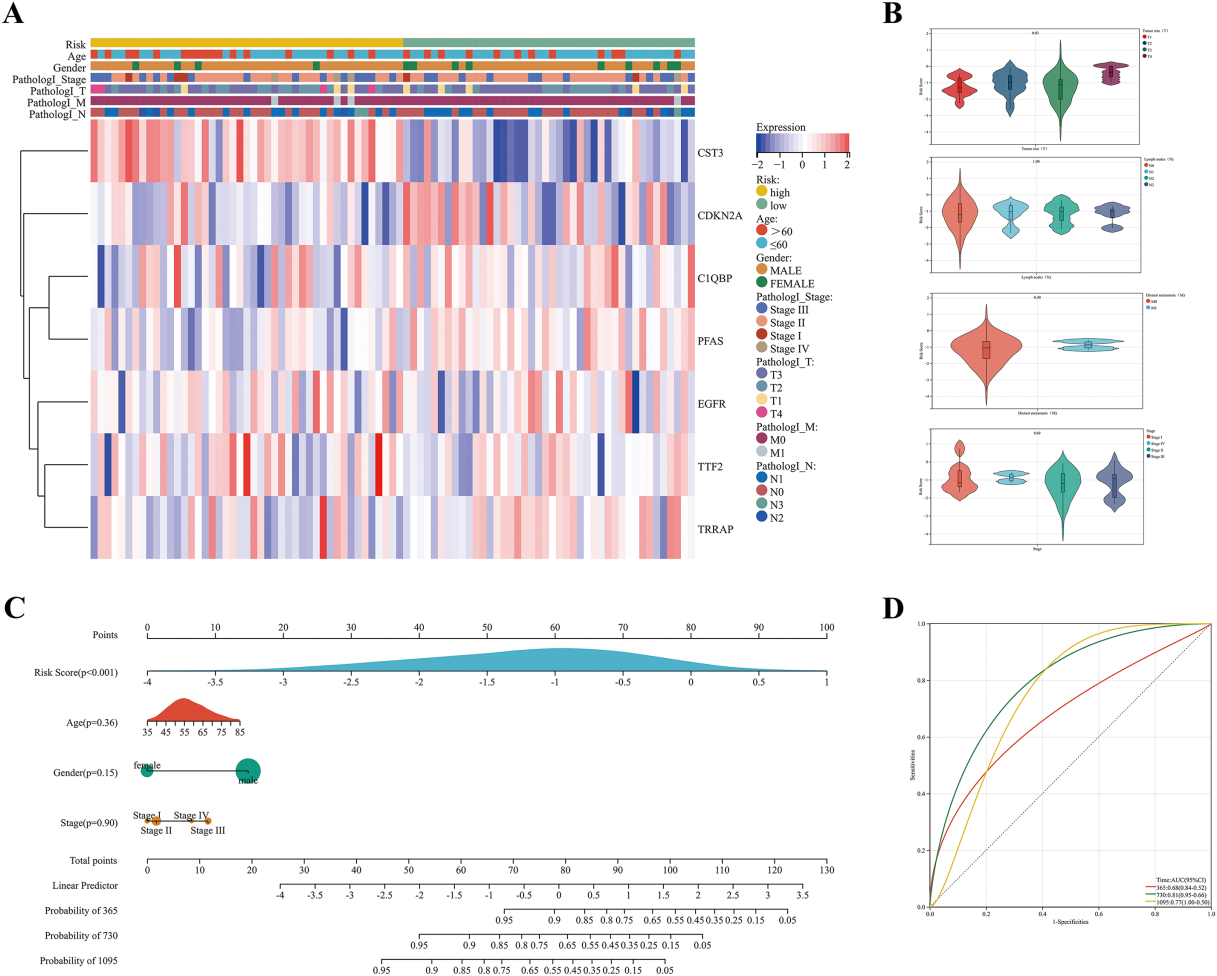
The performance of the prognostic risk model. **(A)** Complex heatmap of the expression levels of the prognostic genes in the high- and low-risk group. Clinicopathologic characteristics, including the age, gender, tumor stage, and pathology stage of T, N, and M are presented above the complex heatmap. **(B)** Correlation analysis of risk score and clinical traits. **(C)** Construction of nomogram using indicated variables to predict individuals’ probability of survival. **(D)** The time-dependent ROC curve of the performance of the prognostic model at 1, 2, and 3 years. The AUC represents the area under the curve.

The expression levels of seven DE-CCGs are shown in the heatmap (**Figure 3A**). The violin diagram manifested that risk scores were significantly related to T category (P = 0.03), but not related to TNM stage, N category, and M category (**Figure 3B**). Age, gender, stage, and risk score were incorporated to construct a prognostic prediction nomogram, which showed that the risk score had the greatest impact on the prediction performance (**Figure 3C**; P < 0.001). The areas under the time-dependent ROC curves (AUCs) for DE-CCGs risk score were 0.68, 0.81, and 0.77 for 1-, 2- and 3-year OS, respectively (**Figure 3D**). The prognostic value of each candidate core gene in TCGA-ESCC indicated their potential as critical prognostic factors in tumors (**Supplementary Figure 4**). The AUCs values in the validation sets further demonstrated the prognostic predictive capability of this model (**Supplementary Figure 5**). Together, the CCGs-related prognostic model demonstrated robust efficiency in predicting OS.

### GSEA for HR groups

**Figure 4:**
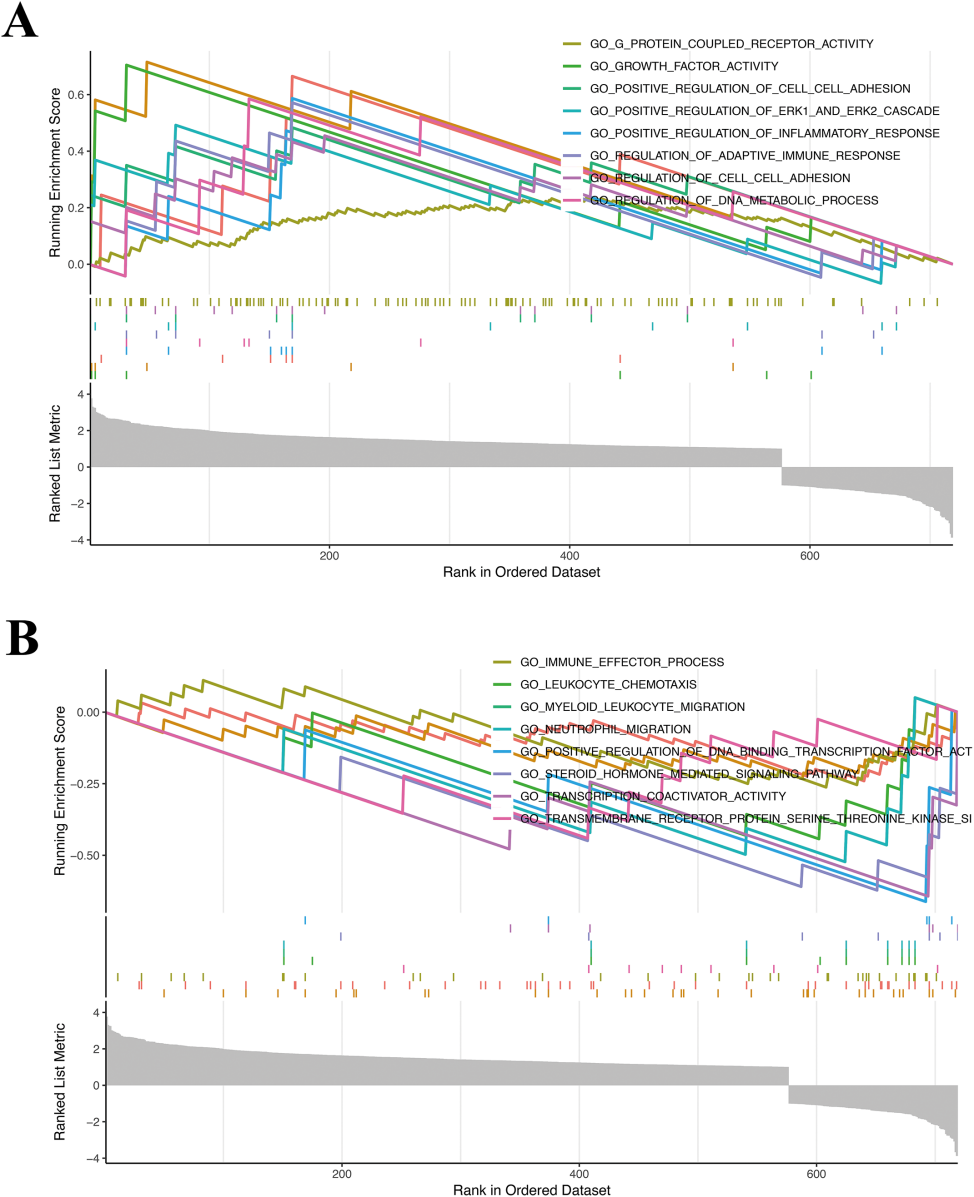
GSEA. The GSEA findings revealed upregulated pathways **(A)** and downregulated pathways **(B)** in the HR group.

GSEA noted that in GO terms, genes in HR groups were markedly enriched for upregulated pathways in immune-linked biological systems, like positive regulation of inflammatory response and regulation of adoptive immune response (**Figure 4A**), while down-regulated pathways comprised immune effector process, leukocyte chemotaxis and myeloid leukocyte migration (**Figure 4B**). These results might unveil the mechanism of genes in HR groups in immune status.

### Association of the risk model with TIICs

**Figure 5:**
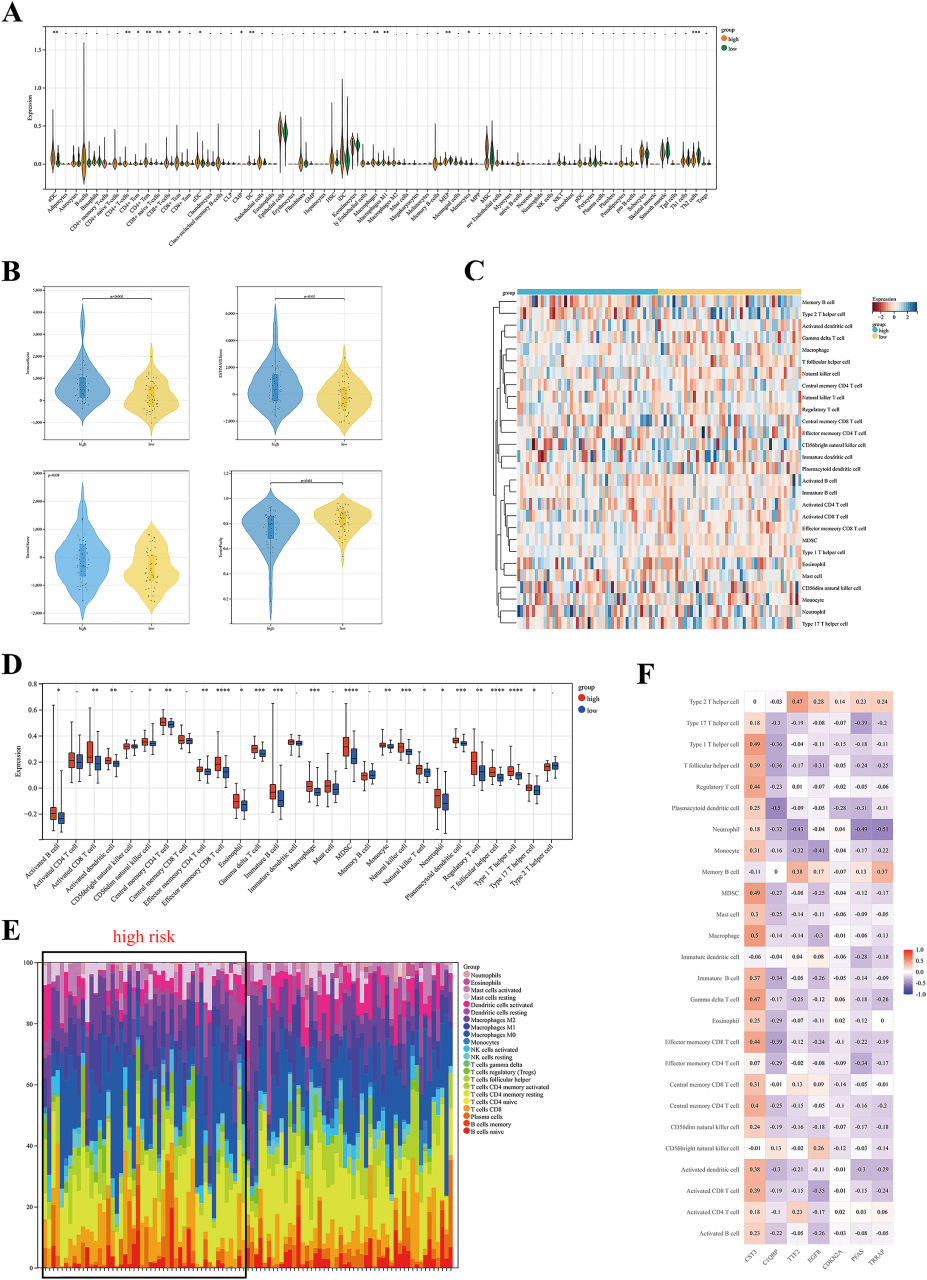
Immune cell infiltration between HR and LR groups. **(A)** Box plot of 64 immune cell types, and corresponding abundance calculated by xCell algorithm. **(B)** Violin diagram of the difference stromal and immune scores access by ESTIMATE algorithm. **(C, D)** Immune infiltration landscape analyzed by ssGSEA score‐based method between the two groups; **(C)** Heatmap of the immune cell types; **(D)** Box plot of the immune cell types. **(E, F)** Immune infiltration patterns analyzed by the CIBERSORT algorithm; **(E)** The bar plot provides an overview of the distribution of immune cells; **(F)** Heatmap shows the correlation of the immune cell infiltration with the prognostic genes. *p < 0.05, **p < 0.01, ***p < 0.001, ****p < 0.0001; ns, no significance.

**Figure 6:**
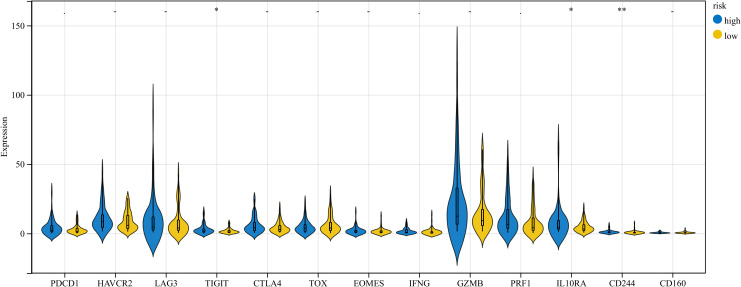
Analysis of immune-related genes in HR and LR groups.

Immune cell infiltration is correlated with prognosis of cancer patients, potentially serving as therapeutic targets to improve patient prognosis (19, 20). Given the prominent importance of immune-related pathways with DE -CCG risk signature identified in GSEA (**Figure 4**), we performed different immunological analyses of the risk model with TIICs. The xCell algorithm manifested that the HR group had higher concentrations of immune cells, such as CD4+ T cell, CD4+Tcm, CD4+Tem, CD8+ T cell, CD8+Tcm, macrophages, and macrophages M1 than the LR group (**Figure 5A**). Next, the ESTIMATE algorithm manifested elevated immune scores (p < 0.001) in HR cohorts (**Figure 5B**). Additionally, the ssGSEA algorithm uncovered a significant correlation of the HR cohort with increased tumor infiltration levels in ESCC. The specific infiltration of immune cell populations is visualized in the heatmap (**Figure 5C**). The Box plot indicated that most immune cells were greatly enriched in the HR group (**Figure 5D**). Moreover, the CIBERSORT algorithm estimated the relative proportions of immune cell subsets. Bar plot illustrated the distribution of immune cells to investigate the relationship between TIICs and risk groups (**Figure 5E**). Notably, the CST3 prognostic gene was highly positively linked with most immune cells (**Figure 5F**). We further analyzed immune regulatory profiles across risk stratifications and found that the HR group exhibited elevated expression of TIGIT, CD244, and IL10RA genes, which may synergistically remodel the tumor immune microenvironment by co-modulating immune checkpoint signaling and effector lymphocyte function (**Figure 6**). This observation not only identifies the distinct immune dysfunction signature of the HR population but also provides a mechanistic rationale for its poor clinical prognosis.

### Tumor immune infiltration analysis and drug sensitivity analysis of CST3

**Figure 7:**
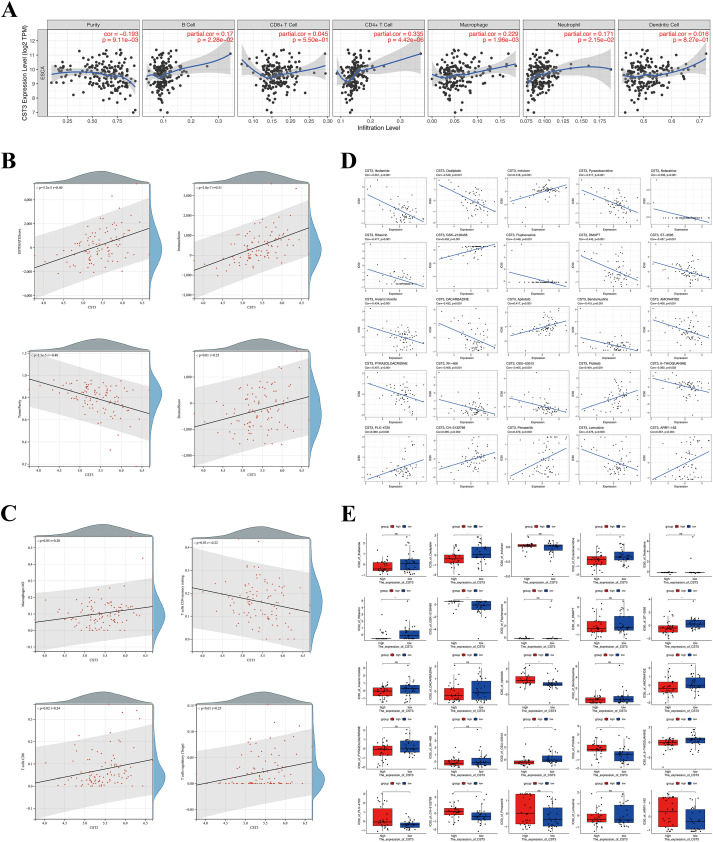
Tumor immune infiltration analysis and drug sensitivity analysis of CST3 in ESCC. **(A)** Correlations between CST3 expression and infiltrating levels of six immune cells in TIMER2.0. **(B, C)** Immune cell infiltration analysis of CST3 expression using ESTIMATE and CIBERSORT algorithm; **(B)** Scatter diagrams analyzed by ESTIMATE algorithm; **(C)** Scatter diagrams analyzed by CIBERSORT algorithm. **(D, E)** Drug sensitivity analysis of CST3; **(D)** Scatter diagrams depicting the relationship between CST3 expression and IC50; **(E)** Box plots showing the IC50 between CST3 high and low expression groups. *p < 0.05, **p < 0.01; ns, no significance.

As CST3 demonstrated a robust correlation with immune infiltration, its involvement in the TME of ESCC was further studied. TIMER2.0 elicited that CST3 expression was positively linked with B cells (r = 0.17, p = 2.28e−02), CD4+ T cells (r = 0.335, p = 4.42e−06), neutrophils (r = 0.171, p = 2.15e−02), and macrophages (r = 0.229, p = 1.96e−03), but negatively correlated (r = −0.193, p = 9.11e−03) with tumor purity (**Figure 7A**). Additionally, the ESTIMATE algorithm showed that CST3 expression was positively linked with stromal score (r = 0.25, p = 0.01) and immune score (r = 0.51, p = 2.0e−07) (**Figures 7B**), which implied heightened complexity in the TME, possibly promoting tumor malignancy. Moreover, CIBERSORT indicated that CST3 expression was positively linked with CD8 T cells (r = 0.24, p = 0.02), resting memory CD4 T cells (r = 0.22, p = 0.03), regulatory T cells (r = 0.25, p = 0.01), and macrophages M2 (r = 0.20, p = 0.05) (**Figure 7C**). Collectively, these findings highlighted the involvements of CST3 in immune status and its potential value in immunotherapy.

Additionally, we tried to screen potential agents with higher drug sensitivity for high CST3 expression patients. The results showed CST3 expression was positively linked with the sensitivity of PI3K/mTOR inhibitors (GSK-2126458, Apitolisib, Pictilisib, and CH-5132799), MEK inhibitors (Pimasertib and ARRY-162), Irofulven, and PLX-4720 (**Figures 7D–E**). Since the monotherapeutic clinical efficacy of PI3K/mTOR and MEK inhibitors is limited in ESCC, they are not yet used in clinical practice for ESCC treatment. However, multiple PI3K/mTOR and MEK inhibitors have demonstrated significant anti-tumor effects *in vitro* and *in vivo* in ESCC (21, 22). Therefore, new drugs or multi-drug combinations should be vigorously developed. And combining CST3 inhibitors with pathway-specific targeted agents may benefit ESCC therapy.

### Association of CST3 and PD-L1 expression with outcomes of patients with ESCC

**Figure 8:**
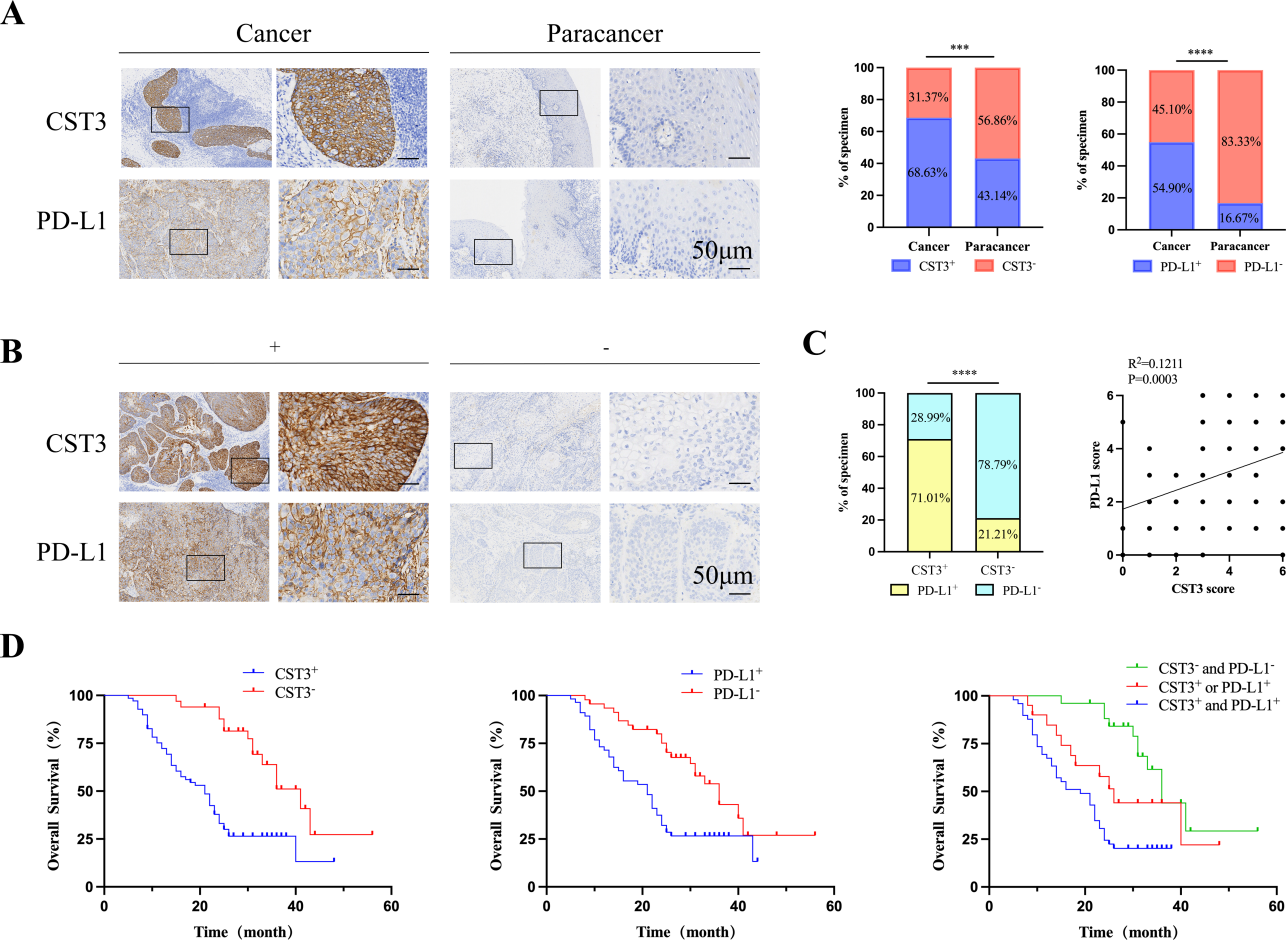
The high expression of both CST3 and PD-L1 is associated with patient with poor ESCC prognosis. **(A)** Expression levels of CST3 and PD-L1 in the ESCC tissues and paracancer normal tissues. Representative IHC images are shown on the left, and statistical analysis is presented on the right (P <0.005, x^2^ test). Scale bars = 50μm. **(B)** Representative IHC images of positive and negative expression of CST3 and PD-L1 in the ESCC tissues. Scale bars = 50μm. **(C)** Statistical analysis of the correlation between CST3 and PD-L1 expression (p =0.0003, Spearman correlation test). **(D)** Kaplan-Meier survival analysis of overall survival for 102 patients based on expression of CST3, PD-L1, or both proteins. ***P < 0.001; ****p < 0.0001; ns, no significance.

Recently, immune checkpoint blockade through anti-PD-1/PD-L1 antibodies has achieved clinical benefits and altered the treatment options for ESCC (23). Considering CST3 is highly correlated with immune cells, we examined CST3 and PD-L1 expression in surgically collected ESCC samples and adjacent normal tissues from 102 ESCC patients. IHC noted that both CST3 and PD-L1 were notably higher in cancer tissues (**Figure 8A**) and were positively correlated (**Figures 8B–C**; R^2^ = 0.1211, p = 0.0003), suggesting that they may be synergistically involved in ESCC development. Moreover, CST3 protein expression was substantially linked with T stage (P = 0.0013), tumor size (P <0.0001), and lymph node metastasis (P = 0.0187) (**Supplementary Table 1**). Survival analysis showed that patients with high levels of both CST3 and PD-L1 had a shorter OS relative to patients with high levels of either protein alone (**Figure 8D**). Collectively, these results indicate the clinical value of CST3 in ESCC and a combination of CST3 and PD-L1 could be considered as an important marker for ESCC prognosis.

### CST3 and PD-L1 synergistically promote ESCC cell growth *in vitro*

**Figure 9:**
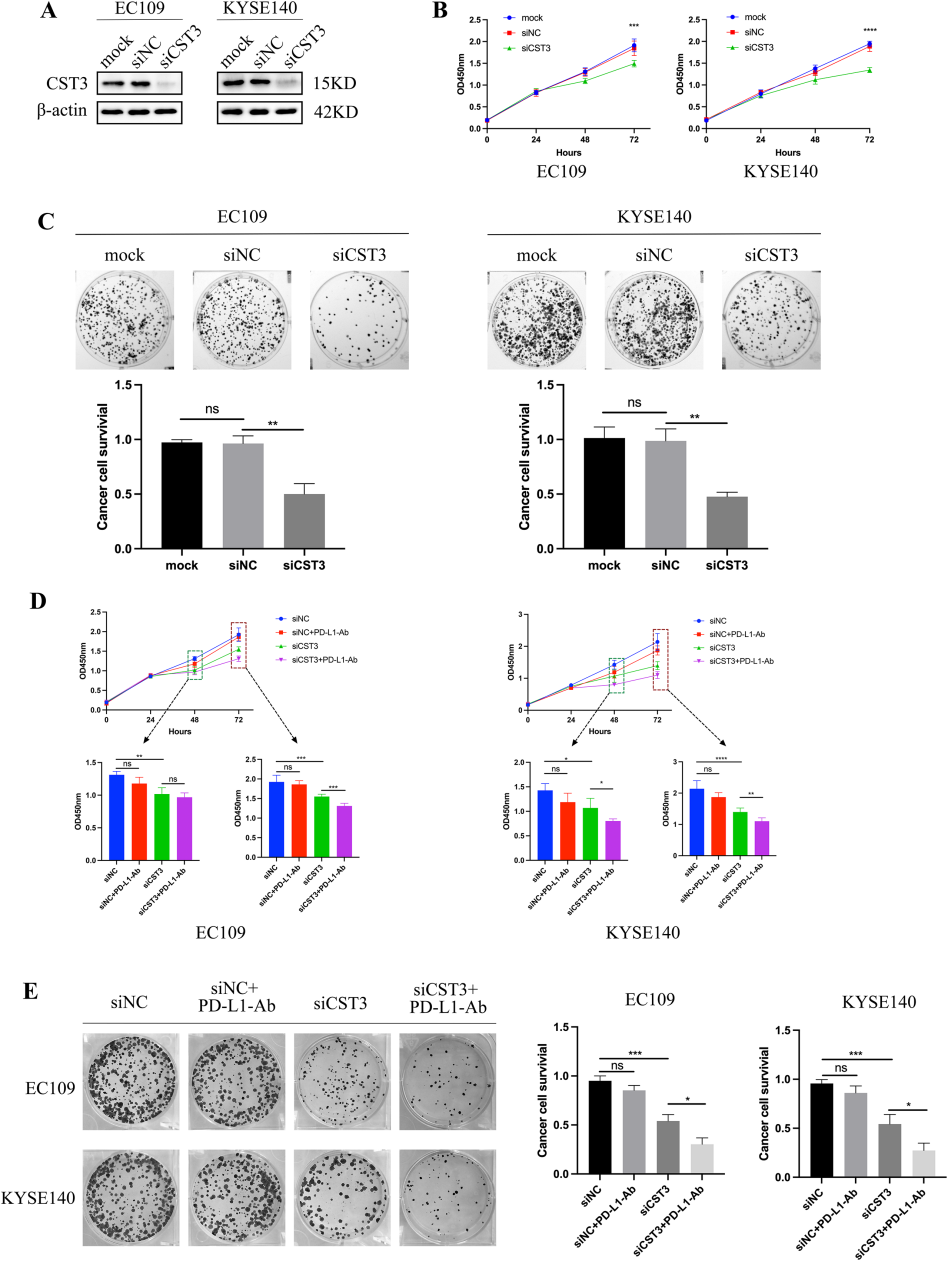
CST3 and PD-L1 are correlated with tumor progression in ESCC. **(A)** Western blot showing CST3 expression in cancer cells transiently transfected with siRNAs against CST3 and their control cells. CCK-8 **(B)** and plate clone formation assay **(C)** show that CST3 promotes ESCC cell growth. CCK-8 **(D)** and plate clone formation assay **(E)** show the proliferation of siCST3-ESCC cells, and their control cells treated with PD-L1-Ab. *P < 0.05; **P < 0.01; ***P < 0.001; ****p < 0.0001; ns, no significance.

To demonstrate the biological function of CST3 in ESCC cells, EC109 and KYSE140 cell lines with CST3 knockdown were successfully established (**Figure 9A**). The proliferation and colony formation assays demonstrated that CST3 knockdown significantly inhibited proliferation of ESCC cell *in vitro* (**Figures 9B–C**). Concomitantly, treatment with a PD-L1-neutralizing antibody to block endogenous PD-L1 activity revealed that concurrent inhibition of CST3 and PD-L1 exerted a synergistic suppressive effect on ESCC cell proliferation (**Figures 9D–E**). Collectively, these results indicate that CST3 exerts a pro-tumorigenic effect in ESCC *in vitro*, supporting its potential as a therapeutic target for ESCC and possibly other malignancies. Furthermore, co-targeting CST3 and PD-L1 may represent a novel therapeutic strategy for ESCC treatment.

## Discussion

ESCC patients have poorer prognoses regardless of chemotherapy, radiation, or immune checkpoint inhibitors owing to the paucity of targeted therapies based on particular driver genes (24, 25). Growing evidence has stated that tumorigenesis is significantly influenced by circadian mechanisms (6, 26). And CCGs in ESCC patients can serve as prognostic biomarkers (27). Immune cells in the TME are crucial in tumorigenesis. Antitumor immune cells initially target and eliminate cancer cells, but cancer cells ultimately evade immune surveillance and limit the cytotoxic effects of these immune cells via various mechanisms (28). Emerging evidence reveals that circadian clocks exert indispensable regulatory effects on cancer initiation and progression, primarily by remodeling the tumor immune microenvironment and modulating the expression of immune-related genes (9). To date, no CCGs-related prognostic indicators have been identified concerning tumor progression and immune infiltration in ESCC. Here, from a holistic view of cancer biology, we focused on tumor progression and the TME to enhance our knowledge of ESCC.

Through comprehensive analyses using WGCNA and machine learning, seven CCGs (CST3, C1QBP, TTF2, EGFR, CDKN2A, PFAS, and TRRAP) that were strongly associated with ESCC were discerned, and a prognostic risk model was built based on these seven genes. Among them, CST3 were associated with poor outcomes in ESCC patients, whereas C1QBP, TTF2, EGFR, CDKN2A, PFAS, and TRRAP served as positive prognostic markers. Kaplan–Meier analysis noted that LR cohorts had considerably higher OS, and ROC curve demonstrated that the model effectively predicted OS of ESCC patients, with those in the HR group exhibiting decreased OS rates and worse prognoses. Besides, HR patients had greatly increased levels of TIICs. And this robust intertumoral activation of leukocytes was accompanied by the expression of T-cells exhaustion markers, such as TIGIT, which is a canonical hallmark of immune escape in various disease settings. This finding suggests that reversing the immunosuppressive microenvironment and restoring the tumoricidal activity of immune cells through strategies such as targeted combination inhibition may significantly improve the clinical prognosis of high-risk populations.

Previous studies have characterized the biological roles and expression profiles of these model genes. Cystatin C (Cyst C), expressed by the CST3 gene, are potent inhibitors of C1 cysteine proteases that control several physiological and pathological processes (29). It was confirmed that the CST3 enhances the migration and invasiveness of specific cancer subtypes, such as lung squamous cell carcinoma (30), triple-negative breast cancer (31), and EC (32). Growing evidence have underscored that CST3 regulates tumor progression by modulating the tumor immune microenvironment (33).

Complement C1q binding protein (C1QBP), also known as p32 or HABP1, is a multicompartmental protein that orchestrates multiple biological processes, including cellular energy homeostasis, mitochondrial morphology regulation and apoptotic signaling pathways (34). Beyond its metabolic roles, C1QBP also serves as a critical modulator of immune and inflammatory responses, through its capacity to integrate diverse signaling cascades (35). Recent findings suggested that C1QBP deficiency disrupts T cell-mediated immune surveillance, ultimately weakening the host’s antitumor immune defense (36).

Transcription Termination Factor 2 (TTF2) encodes a member of the SWI2/SNF2 family of proteins, which play a critical role in altering protein-DNA interactions. TTF2 was overexpressed in almost all solid tumors and correlated with poor survival in patients with several kinds of solid tumors (37).

Epidermal growth factor receptor (EGFR), a canonical ErbB/HER family receptor, is a well-established regulator of cell proliferation, differentiation, and motility (38). Emerging evidence demonstrated that EGFR alterations remodel the immune landscape of the TME by promoting the recruitment of immunosuppressive cells (e.g., M2-like TAMs, MDSCs, and Tregs), and simultaneously suppressing the activation of T cells and NK cells in glioblastoma (39).

CDKN2A, a pivotal tumor suppressor gene recurrently inactivated across human malignancies, has been extensively investigated for its regulatory role in shaping the tumor immune microenvironment and modulating responses to immune checkpoint inhibitors (ICIs) (40, 41). Recent studies revealed that CDKN2A deficiency promotes ESCC development by inducing malignant transformation and enabling immune escape through immunosuppressive microenvironment formation (42).

Phosphoribosylformylglycinamidine synthase (PFAS) is a critical enzyme in *de novo* synthesis of purine, which is upregulated in multiple cancers and strongly associated with poor prognosis in liver cancer (43). Beyond its role in purine synthesis, PFAS also regulates innate immunity to influence viral pathogenesis. Previous reports have indicated that PFAS suppresses innate immune responses in colorectal cancer by downregulating NF-κB signaling via RIG-I targeting (44).

Transformation/transcription domain-associated protein (TRRAP), a component of chromatin remodeling and transcriptional regulation complexes, regulates phenotypes in colorectal, ovarian, and liver cancers (45–47). Mu B et al. found that deubiquitinated TRRAP drives GBM cell proliferation, migration and M2 macrophage polarization (48).

The correlations identified in this study between the seven circadian rhythm genes and clinical indicators suggest that these genes may exert complex roles in tumor initiation and progression through diverse mechanisms. Differential expression patterns of circadian rhythm genes across biological sex, age, and TNM stage further underscore their importance in growth and oncogenesis.

In the risk model, CST3 was the only oncogene and exhibited strongest correlations with immune cells in ESCC. Therefore, CST3 was selected for further analysis to elucidate the function of CCGs in tumor progression and immune infiltration. Previous studies have shown that CST3 governs tumorigenesis by modulating the tumor immune microenvironment. Jiang X et al. noted that overexpressing CST3 suppresses TGF-β1 expression in M2 macrophages and significantly weakens the colorectal cancer cell migration ability (49). Another study revealed that CST3 is associated with the transformation from cytotoxic T cells to exhausted T cells, which could enhance T cell immune activity in lung adenocarcinoma (50). Additional data reported that CST3 can predict immunotherapy failure of cancer immunotherapy by directing the recruitment of Trem2+ macrophages (33). Our bioinformatics modeling demonstrated that CST3 expression was positively linked with B cells, CD4+ T cells, CD8+ T cells, neutrophils, and macrophages in ESCC. Furthermore, the robust associations observed between CST3 expression levels and infiltration of stromal and immune cells suggest heightened cell heterogeneity within the TME. These observations highlight the role of CST3 as an immunomodulator and pro-inflammatory factor, thus potentially contributing to immune escape in ESCC patients.

Immunosuppressants that target PD-1/PD-L1 are primary medicines for EC therapy; yet, their diagnostic effectiveness remains uncertain due to the nascent stage of research (51). Several clinical trials have elicited that PD-1/PD-L1 inhibitors are not effective as standalone treatments for ESCC due to the intricate immune escape mechanisms (52–55). Notably, CST3 was recently reported to be linked to a lack of response to blocking immune checkpoints, which may contribute to resistance against immune checkpoint inhibitors (ICI) treatment response in cancer (56, 57). This finding provided therapeutic rationale for combining PD-1/PD-L1 blockade and CyC inhibition, proposing a promising strategy to overcome ICI resistance in refractory patients (33). Given the paucity of research investigating the correlation between CST3 and PD-L1 in ESCC, we first examined the link between CST3 and PD-L1 to elucidate the function of CST3 in ESCC immunotherapy. The findings revealed that CST3 and PD-L1 were considerably higher in the cancer tissues than in normal tissues and were positively correlated with unfavorable clinicopathological features in ESCC. Moreover, patients with high expression of both CST3 and PD-L1 had shorter OS relative to patients with high expression of either protein alone, suggesting that the combined expression pattern of CST3 and PD-L1 may serve as a robust prognostic biomarker for ESCC. *In vitro* experiments confirmed that CST3 knockdown markedly suppressed ESCC cell proliferation, which was further enhanced by PD-L1 blockade, indicating potential synergistic tumor-suppressive activity. These findings align with prior research proposing CST3 as a potential molecular target for cancer therapy involving protease inhibitors (58). Collectively, our results demonstrate that CST3 likely drives ESCC progression through remodeling the tumor immune microenvironment, an effect mediated by its synergistic interaction with PD-L1. Notably, the potential role of CST3 in predicting immunotherapy failure requires further experimental validation. Future studies should employ larger sample sizes to comprehensively evaluate their correlation with clinical parameters and elucidate the detailed molecular interactions involved. Expanding research to multi-center clinical cohorts will be essential to validate the prognostic and therapeutic value of combining CST3 and PD-L1 as a potential biomarker panel in ESCC. If confirmed, combined targeting of CST3 and PD-L1 might reverse the immunosuppressive tumor microenvironment and restore immune cell cytotoxicity, offering a novel therapeutic strategy for high-risk ESCC patients.

In summary, our study constructs a risk and prognostic model based on ESCC-specific DE-CCGs, which demonstrate robust efficacy in predicting OS in ESCC and significantly correlate with tumor development and immune cell infiltration. Subsequently, *in vitro* investigations indicate that CST3 may serve as a metric for elucidating the function of cysteine proteinases in ESCC progression and may be a target for cancer treatment based on proteinase inhibitors. We initially propose that CST3 and PD-L1 expressions are correlated in ESCC, which suggests that the combined detection of CST3 and PD-L1 may be useful for prognostic assessment in ESCC. In this context, further studies may elucidate the potential therapeutic significance of CST3 for early diagnosis, prognosis, and prediction of therapy responses in ESCC patients.

## Conclusion

The TME is exceedingly intricate, resulting in a limited percentage of ESCC patients benefiting from PD-1/PD-L1-targeted treatment. Consequently, diverse gene targets, targeted therapeutics, and novel immunoreceptors are essential for ESCC management and precision therapy. Despite extensive studies on CCGs in immunology and cancer biology, these genes remain largely underexplored in the realm of tumor immunity. This study examines CCGs in ESCC development and illustrates their correlations with patient prognosis and immune response. Notably, the findings identify that CST3 and PD-L1 have synergistic effects in ESCC and co-targeting CST3 and PD-L1 may represent a novel strategy for ESCC treatment. These novel insights enhance our understanding of the TME and may unveil new immune and therapeutic targets.

## Data Availability

The raw data supporting the conclusions of this article will be made available by the authors, without undue reservation.

## Supplementary material

Supplementary Figure 1 Correlation between trait genes.

Supplementary Figure 2 The prognostic values of each DE-CCG in TCGA-ESCC. **(A)** CST3; **(B)** C1QBP; **(C)** TTF2; **(D)** EGFR; **(E)** CDKN2A; **(F)** PFAS; **(G)** TRRAP. *P < 0.05; **P < 0.01; ***P < 0.001; ns, no significance.

Supplementary Figure 3 Validation of the CCGs-related prognostic risk model in the GSE53625 cohort. (A) The distribution of risk scores, survival status and gene expression profiles for the different risk groups. (B) OS of the two risk groups.

Supplementary Figure 4 The time-dependent ROC curve of the performance of prognostic genes at 1, 2, and 3 years in TCGA-ESCC. **(A)** CST3; **(B)** C1QBP; **(C)** TTF2; **(D)** EGFR; **(E)** CDKN2A; **(F)** PFAS; **(G)** TRRAP.

Supplementary Figure 5 The time-dependent ROC curve in the GSE53625 cohort confirms the predictive efficiency of the prognostic signature.
