# Supplementary figures and images for: Construction of circadian clock signature for tumor microenvironment in predicting survival of esophageal squamous cell carcinoma
Source: Front Immunol. 2026 Feb 12;17:1738892. doi: 10.3389/fimmu.2026.1738892 (PMC12935968; doi:10.3389/fimmu.2026.1738892)

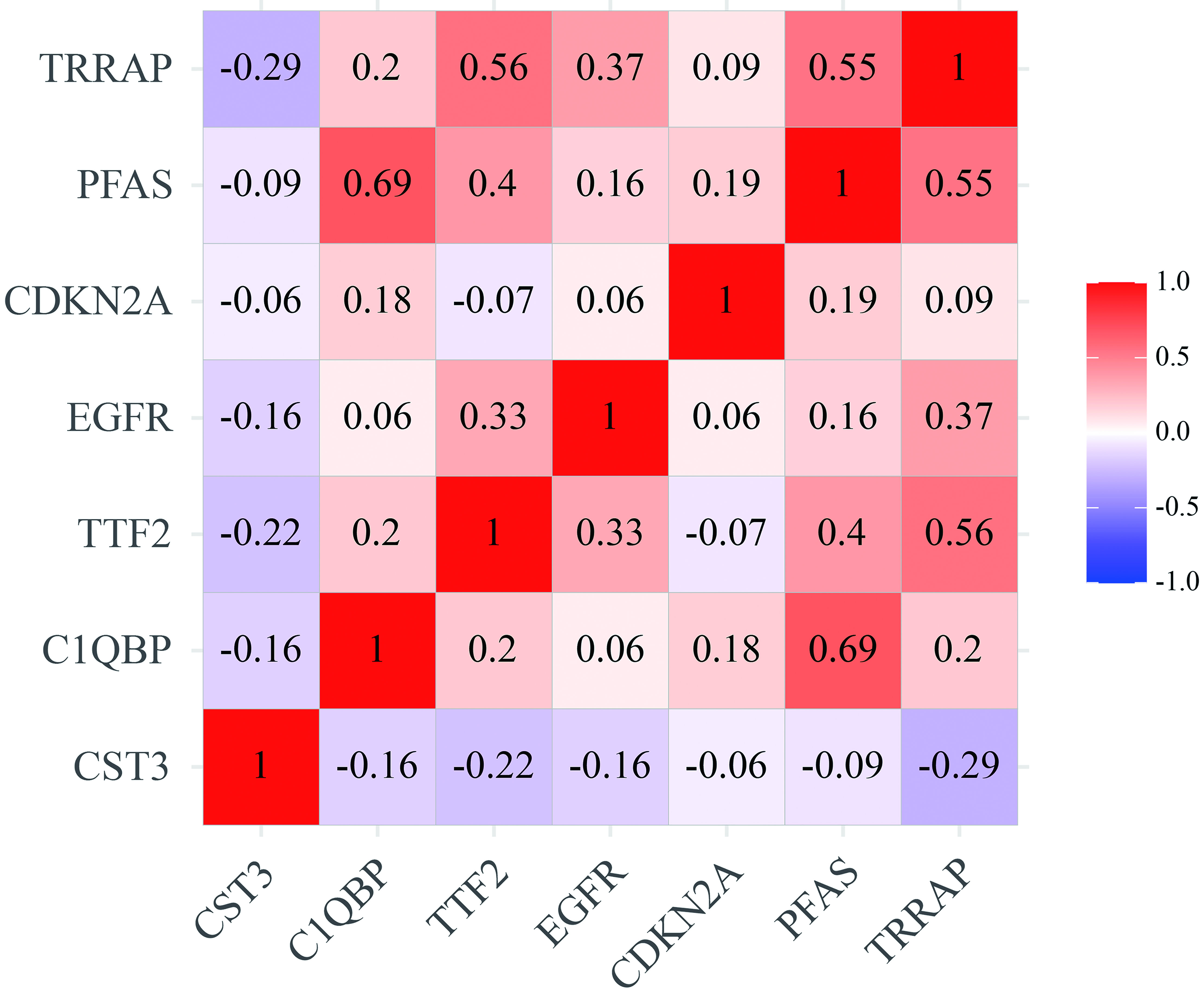

Supplement: Supplementary Figure 1 — Correlation between trait genes. [file Image1.tif]

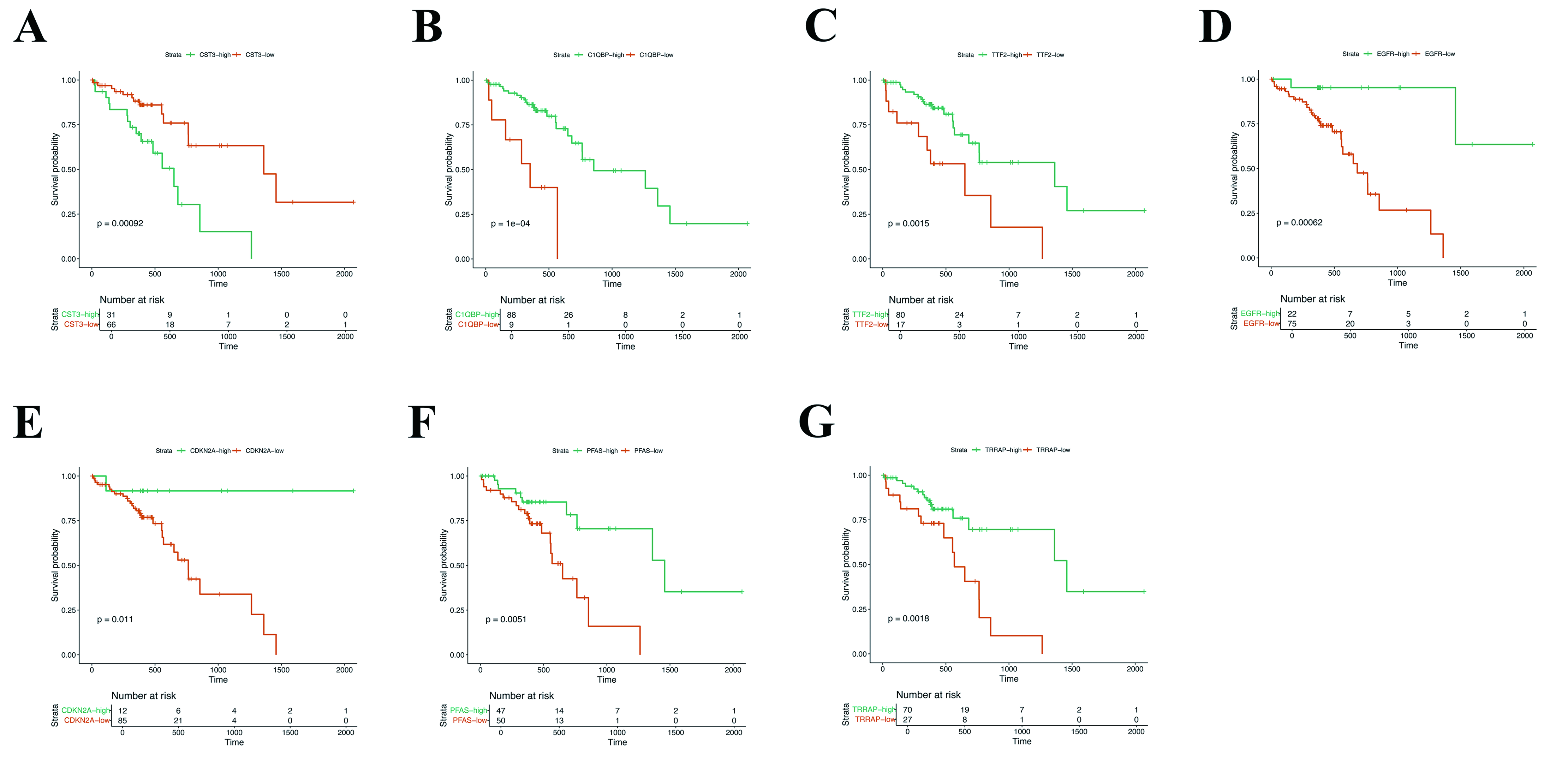

Supplement: Supplementary Figure 2 — The prognostic values of each DE-CCG in TCGA-ESCC. (A) CST3; (B) C1QBP; (C) TTF2; (D) EGFR; (E) CDKN2A; (F) PFAS; (G) TRRAP. *P < 0.05; **P < 0.01; ***P < 0.001; ns, no significance. [file Image2.tif]

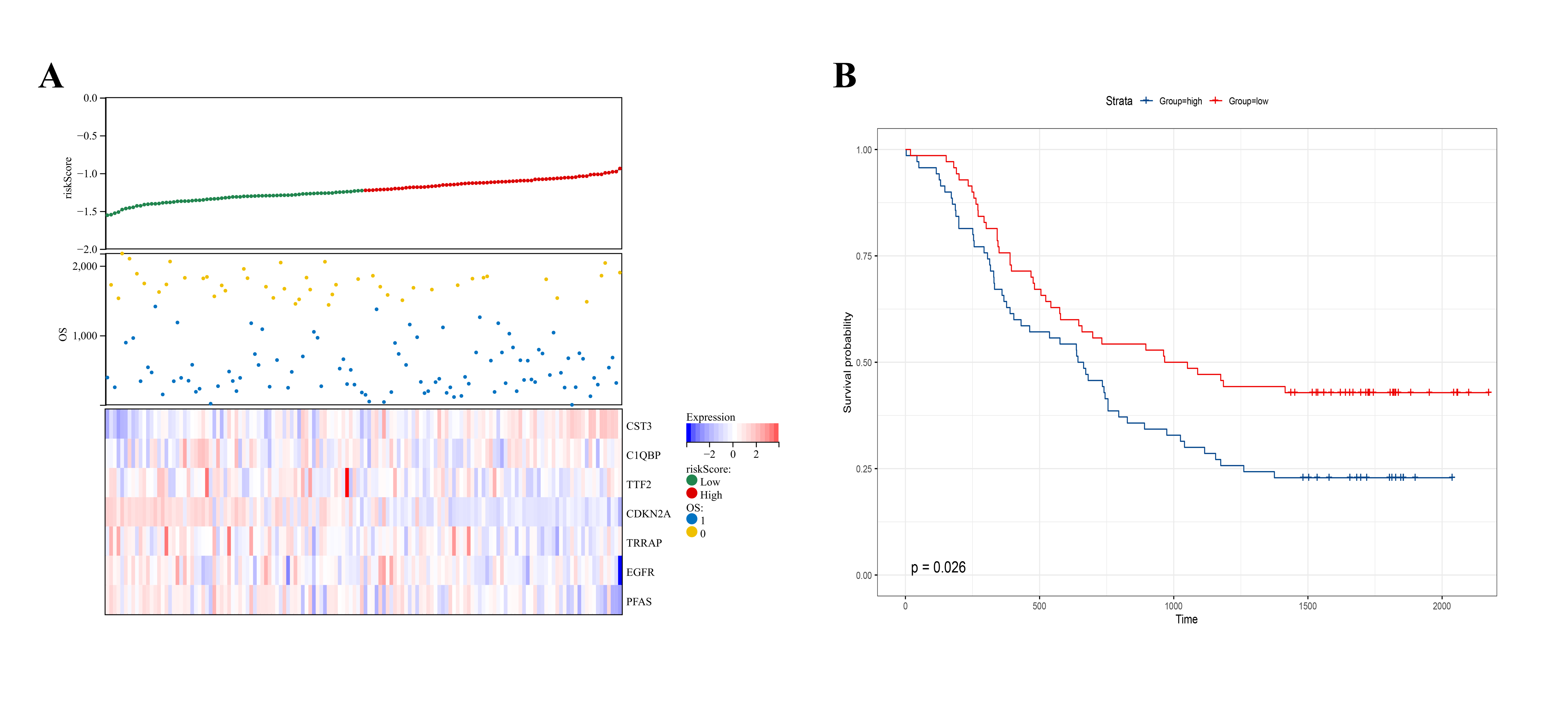

Supplement: Supplementary Figure 3 — Validation of the CCGs-related prognostic risk model in the GSE53625 cohort. (A) The distribution of risk scores, survival status and gene expression profiles for the different risk groups. (B) OS of the two risk groups. [file Image3.tif]

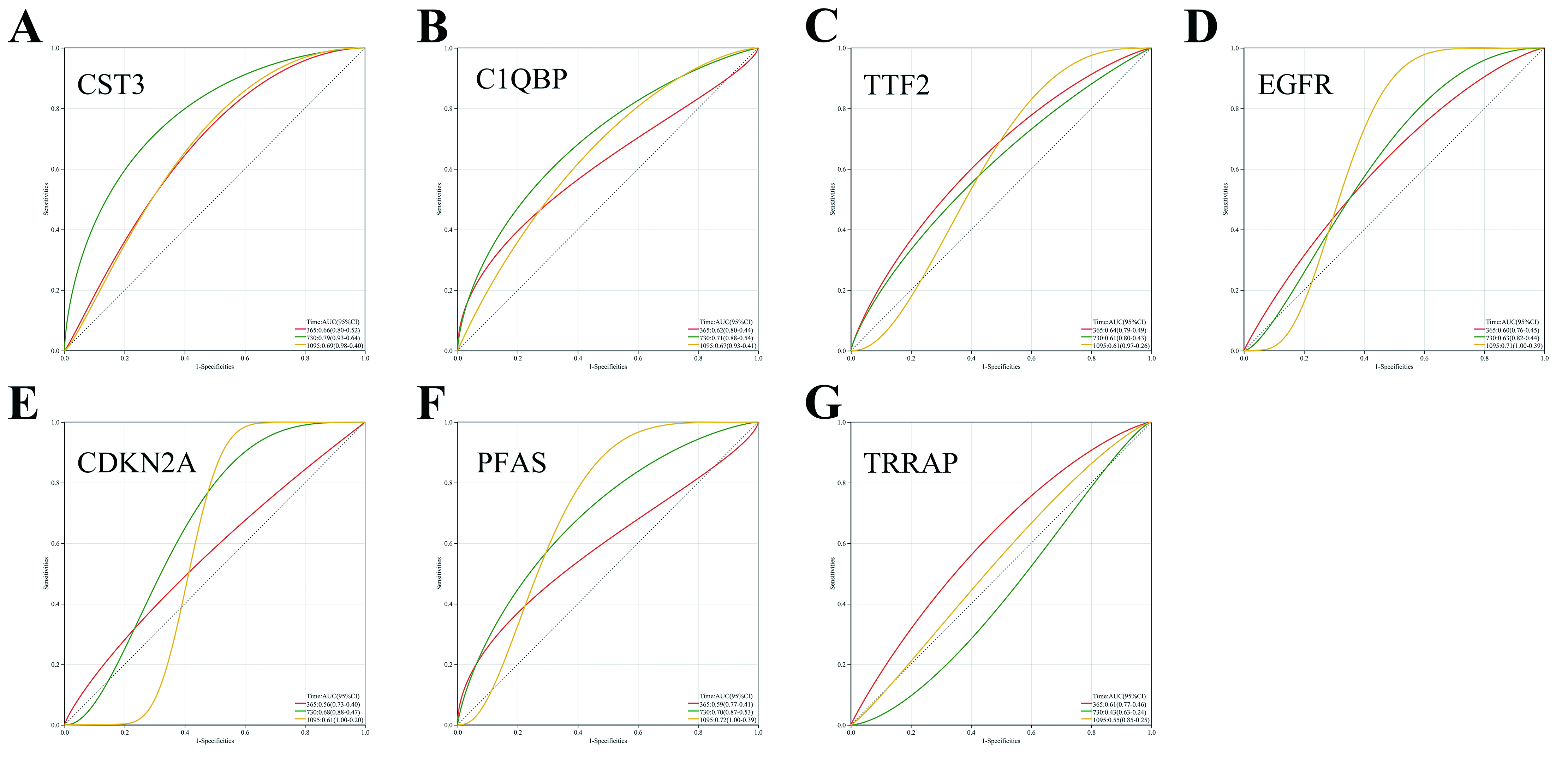

Supplement: Supplementary Figure 4 — The time-dependent ROC curve of the performance of prognostic genes at 1, 2, and 3 years in TCGA-ESCC. (A) CST3; (B) C1QBP; (C) TTF2; (D) EGFR; (E) CDKN2A; (F) PFAS; (G) TRRAP. [file Image4.tif]

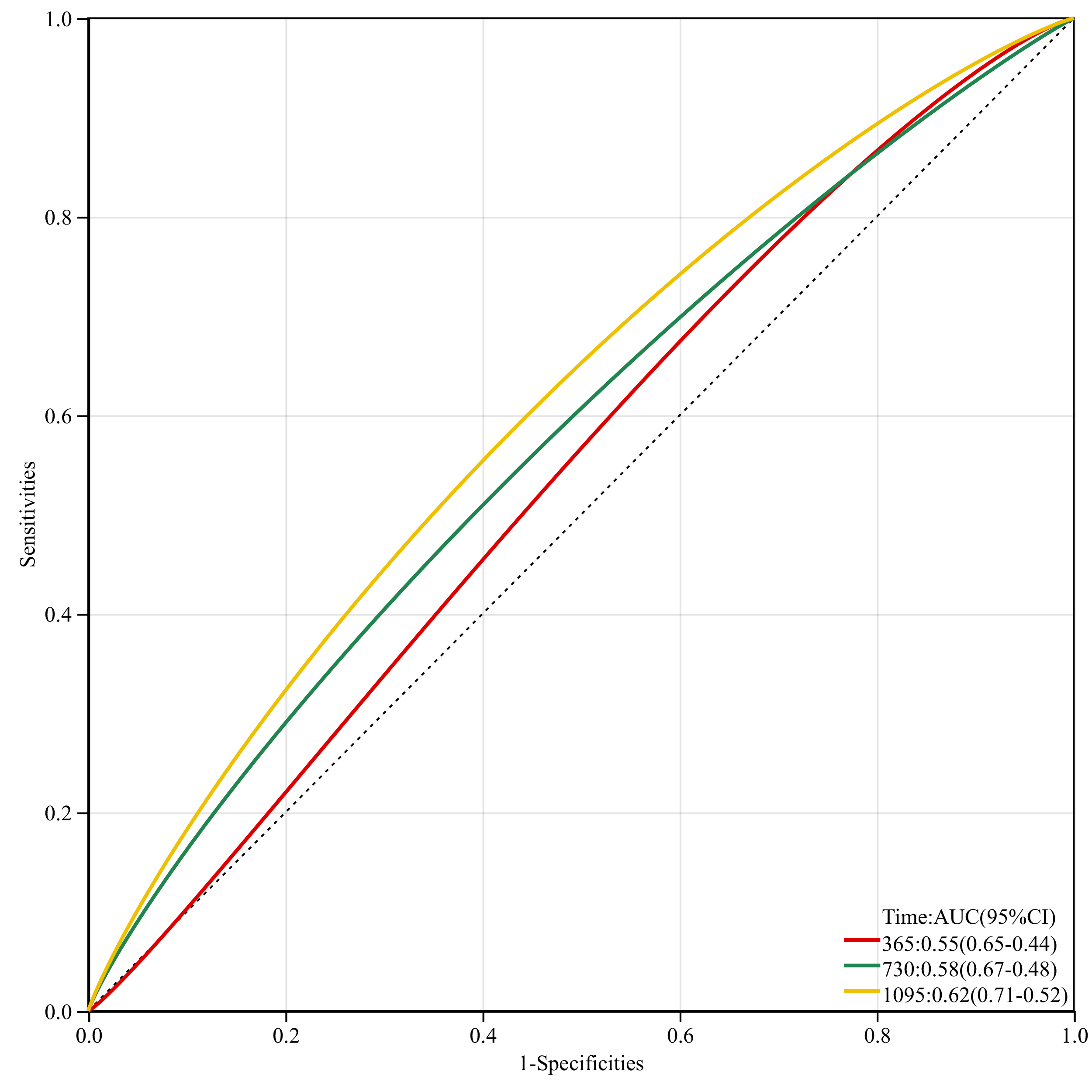

Supplement: Supplementary Figure 5 — The time-dependent ROC curve in the GSE53625 cohort confirms the predictive efficiency of the prognostic signature. [file Image5.tif]
