# Supplementary material for: Construction of circadian clock signature for tumor microenvironment in predicting survival of esophageal squamous cell carcinoma
Source: Front Immunol. 2026 Feb 12;17:1738892. doi: 10.3389/fimmu.2026.1738892 (PMC12935968; doi:10.3389/fimmu.2026.1738892)
Supplement: Supplementary file 6 [file Table1.docx]

**Table 1** Correlation between CST3/PD-L1 expression and clinicopathological parameters in EC patients.

| **Variables** | **CST3** | | **P** | **PD-L1** | | **P** |
| --- | --- | --- | --- | --- | --- | --- |
|  | **+** | **-** |  | **+** | **-** |  |
| Gender | | | | | | |
| Male | 52(50.98%) | 27(26.47%) |  | 43(42.16%) | 36(35.29%) |  |
| Female | 17(16.67%) | 6(5.88%) | 0.6141 | 13(12.75%) | 10(9.80%) | >0.9999 |
| Age（years） | | | | | | |
| ≤60 | 29(28.43%) | 11(10.78%) |  | 26(25.49%) | 14(13.73%) |  |
| ＞60 | 40(39.22%) | 22(21.57%) | 0.5162 | 30(29.41%) | 32(31.37%) | 0.1086 |
| Tumor size(cm) | | | | | | |
| ≤3 | 17(16.67%) | 24(23.53%) |  | 17(16.67%) | 24(23.53%) |  |
| ＞3 | 52(50.98%) | 9(8.82%) | <0.0001 | 39(38.23%) | 22(21.57%) | 0.0281 |
| Differentiation | | | | | | |
| High-Medium | 34(33.33%) | 12(11.77%) |  | 26(25.49%) | 20(19.61%) |  |
| Low | 35(34.31%) | 21(20.59%) | 0.2883 | 30(29.41%) | 26(25.49%) | 0.8425 |
| T stage | | | | | | |
| T1+T2 | 24(23.53%) | 23(22.55%) |  | 20(19.61%) | 27(26.47%) |  |
| T3+T4 | 45(44.12%) | 10(9.80%) | 0.0013 | 36(35.29%) | 19(18.63%) | 0.0281 |
| Lymph node metastasis | | | | | | |
| No | 24(23.53%) | 20(19.61%) |  | 19(18.63%) | 25(24.51%) |  |
| Yes | 45(44.12%) | 13(12.74%) | 0.0187 | 37(36.27%) | 21(20.59%) | 0.0460 |
